# China's community-based crisis management model for COVID-19: A zero-tolerance approach

**DOI:** 10.3389/fpubh.2022.880479

**Published:** 2022-07-22

**Authors:** Ziheng Shangguan, Mark Yaolin Wang

**Affiliations:** ^1^School of Business, Hubei University, Wuhan, China; ^2^School of Geography, Earth and Atmospheric Sciences, The University of Melbourne, Melbourne, VIC, Australia; ^3^Asia Institute, The University of Melbourne, Melbourne, VIC, Australia

**Keywords:** COVID-19, CBDM model, CBCM model, zero-tolerance approach, China

## Abstract

At present, the zero-tolerance and co-existence approaches are the two basic concepts used to manage COVID-19. With the increase in vaccination rates and the continuing impact of the pandemic on people's lives, the co-existence approach has become the mainstream global practice. However, its high infection rate is still an inevitable fact. China was the first country to adopt the zero-tolerance approach to deal with COVID-19 and successfully control it. Due to its immediate effects and low infection rates, this approach has been used in China until now. Through the co-operation of the government and community, China has achieved precise regional lockdowns and patient identification. This article uses the CBCM model to interpret how China has achieved its zero-tolerance approach. Finally, the secondary hazards and applicability of China's CBCM model are discussed. This article draws the following conclusions: (1) China's CBCM basically replicates Singapore's crisis management model for SARS. With the co-operation of the community, it achieved universal coverage of prevention, detection and control; (2) Government leadership in dealing with major crises is very important; (3) In addition to relying on the extreme power of the government to realize China's CBCM model, the two major factors of a submissive society and collectivism have played an important role; (4) China's CBCM model is essentially an excessive anti-pandemic strategy.

## Introduction

**Figure 1:**
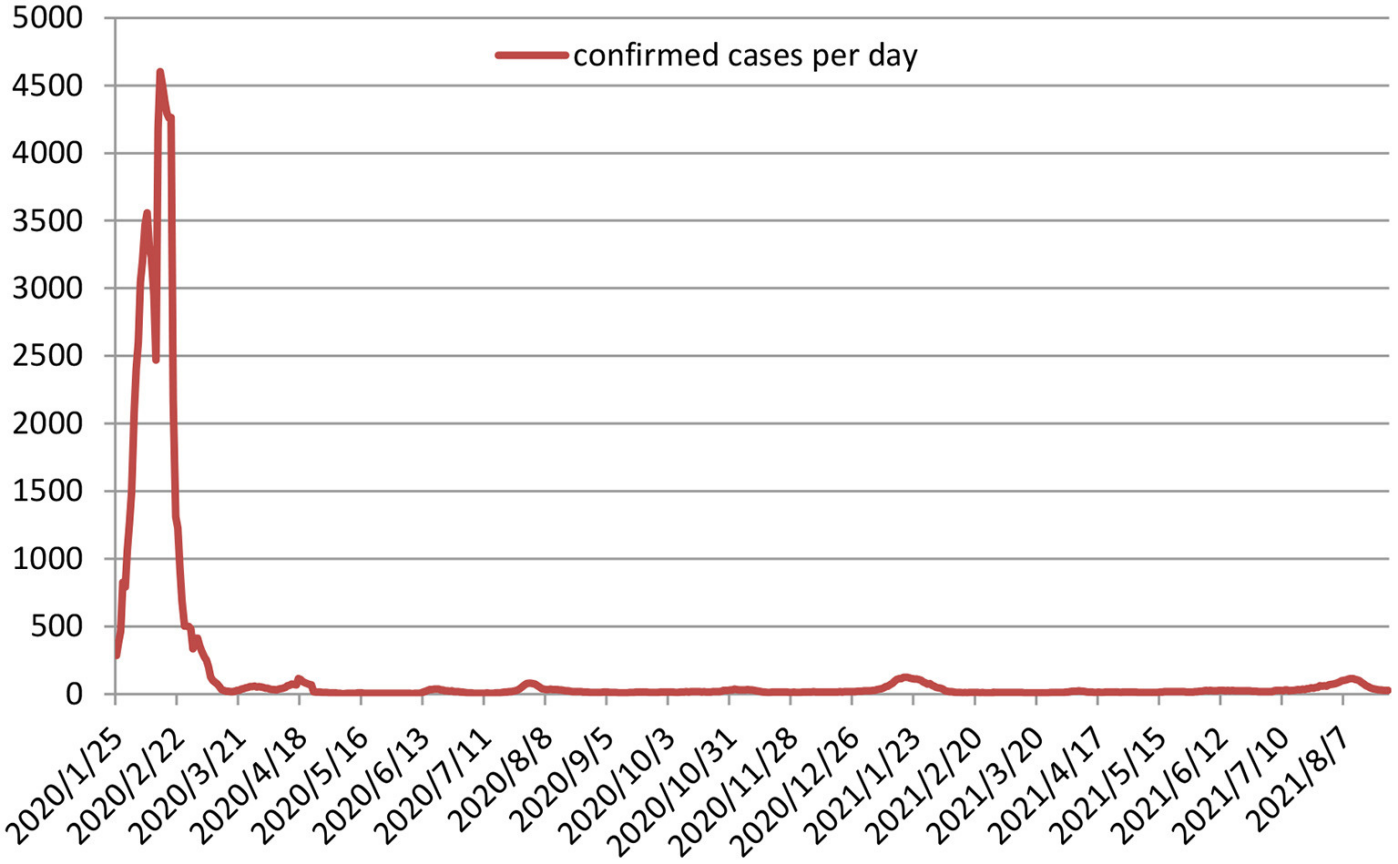
The number of daily confirmed COVID-19 cases in China.

Currently, there has been a global push for COVID-19 vaccinations to create herd immunity. From the perspective of global epidemic prevention and control, China has been relatively successful, although it has experienced problems such as media aphasia, information asymmetry and untimely lockdown of Wuhan in the early stage of COVID-19 pandemic (1). By late August 2021, a total of 122,995 confirmed COVID-19 cases had been reported in China (http://www.nhc.gov.cn/), which ranked 104^th^ in the number of infections worldwide (the higher the case numbers, the higher the ranking). The number of daily cases is shown in Figure 1.

As can be seen from Figure 1, after the Chinese Central Government established the Central Leading Group for Epidemic Response on 25 January 2020, the pandemic was quickly brought under control in just 2 months. Following this, overseas imports were also controlled within 1 month. China has gradually formed a zero-tolerance approach so that once a case is found, the individual and their close contacts are traced and isolated so as to reduce the number of infections to zero. Although this approach cannot result in zero infections, it can help to prevent the spread of COVID-19.

When managing COVID-19, the zero-tolerance and co-existence approaches have been debated by scholars and the media (2). The goal of the zero-tolerance approach is to reduce the number of infections to zero, but it does not mean that it can maintain zero infections. Therefore, there is a time limit in this approach (3). The co-existence approach emphasizes the use of vaccines and effective protective measures (such as wearing masks and maintaining social distance) to keep the infection rate below a certain level, instead of pursuing the complete elimination of the virus (4). China was the first country to adopt the zero-tolerance approach to deal with COVID-19 and was relatively successful in controlling. Since then, countries such as Germany, Italy and Australia have followed this approach. However, the continuous mutation of the COVID-19 virus has caused repeated outbreaks and has affected people's lives, psychology and work to a large extent (5–7). Therefore, some scholars have questioned the zero-tolerance approach. The outbreak of the Delta variant in 2021 has caused many international health experts to regard COVID-19 as an “endemic disease,” and thus prompted many countries to abandon the zero-tolerance approach and switch to the co-existence approach. The widespread use of vaccines worldwide and the increased investment of public health resources have provided a basis for the co-existence approach, but the high infection rate is still an inevitable fact (8).

The zero-tolerance approach has been used in China until now due to its rapid response and low infection rates, and a strong government is an important factor for China to be able to implement it, especially in the early stages of the COVID-19 pandemic. The extreme lockdown that lasted more than 2 months allowed China to quickly control the spread of the virus. However, it is the co-operation of the community that enables the Chinese government to effectively implement the zero-tolerance approach in the later stages. The later stage here refers to the time after 8 May 2020, the first wave of the pandemic was controlled in China (9). Through the deep co-operation between the community and the government, China has achieved a precise regional lockdown and can identify all patients and potential patients as quickly as possible. The basis for this process is a community-based management model. Community is the first responder to the disaster or crisis, and its residents know their own circumstances best, so this community-based management mode emphasizes that residents are the central point in helping to solve the challenges that arise - its essence is a territorial management model (10). This mode can effectively guarantee to meet the needs of vulnerable and high-risk communities the first time, which is difficult for a top-down management mode to achieve (11). However, in the face of large-scale crises, it is not enough to rely solely on the autonomous response of the community, and government intervention is also required (12). Taking advantage of its traditional culture and greater government power, China has successfully practiced a Community-Based Crisis Management (CBCM) model, which is a management model with government as the main body as well as community collaboration. Through this management mode, the number of infections will reduce to zero quickly. This article aims to comprehensively interpret China's CBCM model from the perspective of theoretical research and practical operation.

## Materials and methods

This article mainly uses descriptive and qualitative methods for research. Based on the theories of community-based disaster management (CBDM) and crisis management (CM), this article provides a comprehensive interpretation of China's CBCM, which includes definition of China's community and CBCM, management elements and steps. Based on the management elements and steps of CBCM, this article describes in detail the practical basis and process of China's CBCM. Finally, a qualitative analysis method is used to discuss the secondary hazards and applicability of China's CBCM.

### What is china's CBCM?

#### Definition of china's community

Before introducing CBCM, one of the central concepts of this article will be explained: community. The concept of community was first proposed by German sociologist Tonnies in the 19^th^ century. It refers to a group of people established through blood, neighbors and friends (13). American scholar Gillette pointed out that a community should have six characteristics: (1) in a certain area; (2) residents who interact face to face and have close relationships; (3) common interests; (4) sense of community; (5) organizations formed for long-term co-operation for the benefit of all inhabitants; (6) core interests (14). McMillan and Chavis (1986) put more emphasis on the interaction of members in the community. They identified four elements of “sense of community”: (1) Membership: feeling of belonging or of sharing a sense of personal relatedness; (2) Influence: mattering, making a difference to a group and of the group mattering to its members; (3) Reinforcement: integration and fulfillment of needs; (4) Shared emotional connection (15). James etc. define community very broadly as a group or network of persons who are connected (objectively) to each other by relatively durable social relations that extend beyond immediate genealogical ties and who mutually define that relationship (subjectively) as important to their social identity and social practice (16). In general, a community can be understood as a social group gathered together because of sharing common values or culture, and the group lives in the same area and has direct or indirect continuous interaction.

Obviously, the definitions above are based on the perspective of interpersonal relationships, which are suitable for explaining China's rural communities. Residents of China's urban communities may not fully possess the four elements proposed by McMillan and Chavis (15). Urban community in China is similar to province, city, county and town in administrative division (from large to small), and it is a subdivision of the urban area within the town. The division of urban communities does not depend on the interaction between their residents, but a simple spatial distinction. In addition, the definition of urban community in China pays more attention to their functionality, including: (1) Guarantee function: rescue and protect vulnerable groups in the community; (2) Security and stability function: resolve various social conflicts and ensure the safety of residents' lives and property; (3) Management function: manage the social life affairs of the people living in the community; (4) Service function: provide social services for community residents; (5) Educational function: improve the civilized quality and cultural accomplishment of community members (17). These functions of China's urban communities have given them a major role to perform in the zero-tolerance approach. Rural communities and urban communities do not have administrative functions themselves, and they are the basic unit of grassroots autonomy. But they are subject to the leadership of the village branch and the sub-district office which is the smallest administrative management unit in China. Therefore, it is easy for China to form a management model with the government as the main body as well as community collaboration.

#### Definition of china's CBCM

China's CBCM model is a management model explored in practice in order to reduce the number of infections to zero. Its essence is still a community-based management model. The community-based management model is a territorial management model proposed to overcome the failure of top-down management to deal with risks in time. This management model was originally proposed to help people respond more quickly to natural disasters, such as hurricanes, earthquakes, and floods. Therefore, this management model is also called CBDM (18). With the rise of CBDM research in Asia, CBDM has gradually evolved into Community Based Disaster Risk Management (CBDRM) (10). CBDRM is defined as an inclusive, active and autonomous community driven processes, which aims to solve the driving factors of disaster risk, reduce disaster risk and build social resilience. This management model is community-centric (19).

The concept of CBCM is rarely mentioned in existing CBDM research, because the impact of general natural disasters is difficult to reach the required level of crisis. Normally, natural disasters can be controlled at the community level, but the emergence of crises may affect the entire nation and even the world (20), such as the global health crisis caused by COVID-19 pandemic. Therefore, it is not enough to rely solely on the autonomous management of the community. Government departments also need to carry out unified management and resource scheduling. Just as Birrell emphasized that it is very important for government departments to formulate strategies when facing major crises (21). In combination with relevant research on crisis management and the definition of CBDRM, and taking into account China's cultural and political background, this paper defines China's CBCM as a community-wide process in which the government, ministries, community organizations and community residents jointly deal with the driving factors of crisis, eliminate crisis and monitor crisis. Among them, the government is responsible for formulating a unified epidemic prevention strategy, mobilizing resources and information disclosure, while the community is in charge of implementing government policies according to its own situation. It is worth noting that China's one-party authoritarian system has natural advantages in formulating and implementing a unified response strategy, and its one-size-fits-all governance methods provide an effective foundation for the zero-tolerance approach.

#### Management elements of china's CBCM

Since China's CBCM model relies on the community, the management elements of CBDM are also applicable to China's CBCM. In 2002, the United Nations Center for Regional Development (UNCRD) initiated the “Sustainability in Community Based Disaster Management” research project. Based on the current research, the project summarized three key factors of CBDM: education, cooperation and self-help (22). As the basis of CBDM, the importance of education has been widely accepted (23, 24). The United Nations Development Program (UNDP) pointed out that education involves three aspects: (1) systematic training for community managers (disaster risk management committee (DRMC), related organizations and personnel) and social workers; (2) Popularize disaster prevention and control knowledge; (3) disaster avoidance simulation training for community residents (25). The research of Liu et al. further confirmed the necessity of education and training in CBDM (26). With an educational foundation in place, co-operation should be extensive. Shaw pointed out that the emergence of “outsiders” should be avoided as much as possible in CBDM (27). This is particularly important during COVID-19, as it is evident that the infection rate is lower in countries where mask wearing is more common (28). In addition, full consideration should be given to the ability of vulnerable groups to participate in co-operation. These vulnerable groups include: the elderly, the disabled, children and pregnant women (29, 30). Self-help is the most important part of CBDM and an important manifestation of territorial management. Ekanayake pointed out that evacuation, mutual rescue and material distribution organized by community residents themselves based on situation can effectively reduce casualties when disasters occur (19). The community managers, social workers and volunteers with certain professional skills play an important role in the process. Among them, community managers are responsible for formulating response strategies, while social workers and volunteers are in charge of supervision and implementation (25, 31). However, the management elements of CBDM are not sufficient to achieve the completion of China's CBCM. As mentioned in Section 2.1.2, the role of the government in the crisis needs to be considered. When a crisis breaks out, relying solely on the autonomous management of the community may cause management chaos. Therefore, it is necessary for the government and related departments to formulate a unified response strategy in crisis management (32). Based on the management elements of CBDM and the definition of China's CBCM model, we believe that the management elements of China's CBCM include education, cooperation, self-help, and government management.

#### Management steps: China's CBCM vs. Singapore's CM

Singapore's successful response to SARS provides valuable lessons for other countries to control the spread of COVID-19. Based on CM theory, Tiong summarized steps of CM in Singapore, including (33):

- dealing with feelings and drama associated with a crisis.
- assessing danger and threats within a short time.
- strategic planning and reorganization of the crisis situation.
- mobilization of resources to meet the challenge.
- establishing communication channels and education.
- restoring a new level of equilibrium.

Obviously, these steps are highly dependent on the strong management of government and the extensive cooperation of the public. For China, however, these are inherent advantages. For one thing, the China's authoritarian political system enables the central government to quickly mobilize all levels of government to implement management as soon as possible, which has also been confirmed in the early stage of the pandemic (34). For another thing, China's community management system enables the Chinese government to make good use of the community to penetrate into the grassroots, so as to maximize the coordination of the cooperation (17). In addition to the formulation of the overall epidemic prevention strategy, China's crisis management process can be completed at the smallest cellular unit, that is, the community level. The management steps of China's CBCM can be simplified into the following 5 steps, which are basically the same as Singapore's CM.

- Selecting the Targeted Community.
- Promote Cooperation and Understanding.
- Participatory Crisis Assessment.
- Implementation of Community Management.
- Monitoring & Evaluation.

### Data collection

Data sources for this article include official websites of government departments, documents issued by the government departments, academic journals and media reports.

#### Official websites of government departments

The data sources in this article come from the websites of relevant Chinese government departments, including The Ministry of Education of the People's Republic of China (MOE, http://www.moe.gov.cn), China Watch Institute (CWI, http://www.chinadaily.com.cn), National Health Commission of the People's Republic of China (HC, http://www.nhc.gov.cn), Chinese Center for Disease Control and Prevention (CDC, https://www.~chinacdc.cn).

#### Documents issued by the government departments

These key government documents we reviewed include: “Opinions on punishing crimes that hinder the prevention and control of the COVID-19” jointly issued by the Supreme People's Court, the Supreme People's Procuratorate, the Ministry of Public Security and the Ministry of Justice; “Notice on Further Strengthening the Organization and Management of National Nucleic Acid Testing” issued by the NHC; “Notice on adjusting the price of COVID-19 nucleic acid testing items” issued by National Healthcare Security Administration (NHSA).

#### Academic journals and media articles

In addition, our data sources also include international journals and media articles, such as SSCI journal articles, Xinhuanet, Sina, Netease, Kuaizixun, Stdaily, Voice of America News.

## Results

### The practical basis of china's CBCM

The management elements of CBCM are the foundation of its practice. We pointed out the four elements of CBCM, namely: education, cooperation, community self-help and government overall management. It is with these elements that China can successfully complete the CBCM in the later stage of the epidemic.

#### Education

UNDP puts forward three educational requirements for CBDM. For the later stage of the pandemic, Chinese communities basically meet the requirements. First of all, under the mobilization of the government, universities in various provinces in China have begun to offer emergency management majors and provide relevant training to the society (35). As of 22 July 2020, 21 universities have established undergraduate programs in emergency management, 13 have established master's programs, and 20 have established doctoral programs. Among them, with the support of the Ministry of Education, some universities launched the “Smart Learning Workshop of Emergency Safety (2020)” project with enterprises to train professional management personnel for the community (http://www.moe.gov.cn/). Secondly, students majoring in social work became the main force of volunteers who played a crucial role in assisting community management, including organizing residents to regularly learn about epidemic prevention and control knowledge and supervising community residents to abide by relevant regulations (36). In addition, mainstream media and social platforms actively popularize the pathogenic mechanism and transmission route of COVID-19, and continuously remind people to wear masks, wash hands frequently and keep indoor ventilation (37). Therefore, Chinese communities have professional managers in the later stages of the pandemic, and their residents basically have the knowledge of prevention and control. Although it is difficult to conduct simulation training for community residents on a large scale in mainland China, the practice in the early stages of COVID-19 pandemic provide sufficient management experience to China's communities, which achieves the effect of simulated training to a certain extent.

#### Co-operation

The emergence of “outsiders” will reduce the efficiency of CBCM, especially in the public health crisis caused by COVID-19, so extensive cooperation is required. According to the report of China Watch Institute (CWI), residents in medium-risk and high-risk communities consciously conduct home quarantine, and residents in low-risk communities actively wear masks in some places with high population density (see Table 1 for risk level classification) (36). During the pandemic, Wang et al. conducted a survey on the social attitudes of Chinese people, which showed that 99.2% of the people across the country thought it necessary to reduce travel, and 99.2% of the people thought they should wear masks when going out (38). Similar survey conducted by Shi et al. indicated that 98.71% of the people believe it is necessary to reduce travel (http://www.ringdata.org/article/358.html). Therefore, it can be seen that Chinese people do generally co-operate with the government and the community to a high degree, regardless of their own self-interests.

**Table 1:** Severity classification of the COVID-19 pandemic.

**Risk level**	**Rating criteria**	**Management method**
High risk	The number of existing cases has exceeded 50, and there has been at least one cluster within the past 14 days.	• Prohibit all residents in the high-risk town from leaving their homes • Except for enterprises related to epidemic prevention and control, public utility operations, people's daily necessities, and other important national livelihoods, all work will be suspended
Medium risk	There are new cases within the past 14 days and the number does not exceed 50, or the number exceeds 50 and there is no cluster within the past 14 days.	• Prohibit residents who live in the risk-related communities from leaving their homes • Except for enterprises related to epidemic prevention and control, public utility operations, people's daily necessities, and other important national livelihoods, all work will be suspended in the risk-related communities • Activities in communities that are not suspended in the medium-risk town need to check body temperature, keep the density of people, reduce the gathering of people, strengthen personnel protection, and eliminate hidden risks
Low risk	No existing cases; or no new cases for the past 14 days.	• Open up the actions of the residents in the town • Monitor the epidemic

*Note: (1) The personnel of the pandemic prevention agency, community managers, social workers, and volunteers can move within their work scope under the authorization of the local government*.

*(2) Medium risk management methods can be enhanced according to the actual situation*.

#### Community self-help

Chinese communities strictly implement grid management. Each grid is equipped with community workers who are composed of doctors, neighborhood committee members, security guards and personnel from the civil affairs ministry. They can develop professional emergency management strategies when the virus breaks out. At the same time, a large number of volunteers have also participated in the management of the virus, which provides effective implementation of the strategies. According to CWI statistics, about 4 million community workers and volunteers visited 650,000 urban and rural communities across the country. Their work includes: (1) Disseminating knowledge of epidemic prevention to residents, especially vulnerable groups such as the elderly and the disabled; (2) Visiting residents' home to identify close contacts; (3) Disinfecting and closing public areas; (4) Provide substances for residents in quarantined communities (36).

#### Government management

The government here refers to governments at all levels and their relevant agencies. The former includes the central government, provincial governments and local governments. The latter includes the Centers for Disease Control and Prevention (CDCP) and the Health Commission (HC). Governments at all levels are mainly responsible for the allocation of material, medical and social resources between provinces and cities; CDCP for completing the epidemiological survey (www.chinacdc.cn); HC for formulating and implementing epidemic prevention measures (www.nhc.gov.cn). Their work is coordinated with each other.

In the process of issuing an epidemiological survey, CDCP used big data to track patients and close contacts. Big data mainly comes from government branches, communication enterprise, shopping platforms, social media and payment platforms (39), and the mobilization and integration of these resources are completed by governments at all levels. Government branch mainly includes Civil Aviation Administration (CAA) and Roads and Traffic Authority (RTA); communication enterprise mainly includes China Unicom, China Telecom and China Mobile; shopping platform mainly includes Meituan and Taobao; social and payment platforms mainly include Wechat and Alipay. Among them, government branch, communication enterprise and shopping platform are mainly responsible for collecting personal identification information and geographic location information. In addition to the above functions, social and payment platforms can also conduct questionnaire surveys, identify personal risk levels and display personal vaccination status. With the continuous integration of technology, close contacts that stayed with the patient in a space of 800 m × 800 m for more than 10 minutes can be accurately identified (40).

HC divided the risk levels of the pandemic into three levels, as shown in Table 1. The division is based on the town-level administrative area as the basic unit. When the number of existing cases has exceeded 50 and at least one cluster occurred within the past 14 days, it is considered high risk. Strict lockdown will be implemented for all communities in high-risk areas. When there are new cases within the past 14 days and the number does not exceed 50, or the number exceeds 50 and there is no cluster within the past 14 days, it is regarded as medium risk. Some of the severe communities in medium-risk areas need to be locked down. When there are no existing cases or no new cases for the past 14 days, it is considered low risk. All communities in low-risk areas will be fully open. According to the risk level divided by HC, central government has formulated corresponding management methods, and standards can be enhanced according to the actual situation.

### China's CBCM at work

**Figure 2:**
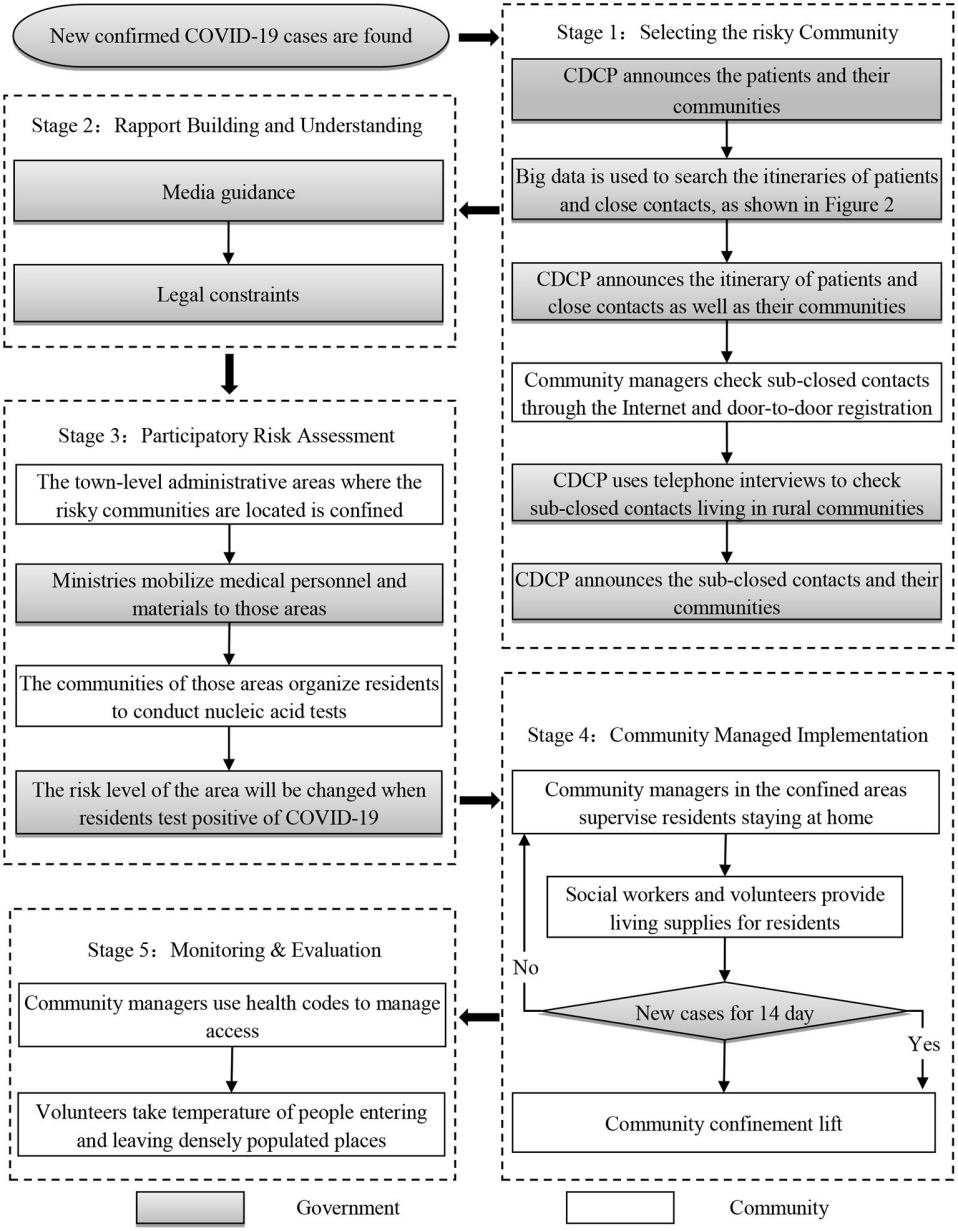
Steps of China's CBCM model.

Figure 2 shows the specific process of China's CBCM, and each steps is described in detail below. By strictly implementing these 5 steps, the zero-tolerance approach is achieved by China.

#### Selecting the targeted community: Track the communities where patients, close contacts and sub-close contacts are located

When new confirmed cases of COVID-19 are found in the hospital, the CDCP will announce their communities on the official website as soon as possible. Then, the CDCP will immediately start an itinerary survey of the patients, while searching for close contacts and their itinerary. After obtaining all aspects of data verification, CDCP will announce the itinerary of patients and close contacts as well as close contacts' communities. After that, each community manager determines whether there are sub-close contacts in the community through network platform or door-to-door registration, and reports to the local CDCP. As for the poorly managed and rural communities, the survey of sub-close contacts was completed by the local CDCP through telephone. The local CDCP will then announce the communities where the sub-close contacts are located. Through the above steps, the communities where patients, close contacts and the sub-close contacts are located are determined. The speed of identifying the target communities is quite rapid. In the case of the Delta virus outbreak at Nanjing Lukou Airport, it took only 10 days for the Nanjing CDCP to identify the target communities after the first case was announced on 20 July 2021 (www.njcdc.cn/).

#### Promote cooperation and understanding: Mass media publicity and legal provisions

Extensive cooperation is based on value recognition and legal constraints. Some scholars believe that the spirit of collectivism is a huge advantage for China to successfully manage the pandemic (41), and media publicity and coverage have become an important means to strengthen collective action among the Chinese population. During the pandemic, smartphone apps such as TikTok, Tencent, and Volcano released numerous videos which showed the hard work of medical staff, social workers and volunteers on the front line (36). Elderly people who don't know how to use smartphone can get relevant information through CCTV News or promotional videos played by community. Publicity is not always helpful because cognitive differences are common. Therefore, there have been legal provisions introduced to help counter media propaganda. On 6 February 2020, four ministries in China, namely: the Supreme People's Court, the Supreme People's Procuratorate, the Ministry of Public Security and the Ministry of Justice, jointly issued the “Opinions on punishing crimes that hinder the prevention and control of the COVID-19.” It clearly stipulates the relevant responsibilities of the Criminal Law and the Public Security Management Law for non-cooperation with pandemic prevention (42). The notice is also posted on the bulletin boards of each community.

#### Participatory crisis assessment: Community nucleic acid testing

Once the community where the patients, close contacts, and sub-close contacts are determined, the town-level administrative area in which the community is located will be locked down, and the number of new cases in the next 14 days will be used to assess the risk level. As described in Table 1. Since everyone in the target community may become a sub-close contact, in order to achieve the purpose of early detection and treatment, the local CDCP will conduct nucleic acid testing for all residents in the town-level administrative area. Once the patients are found, they will be immediately sent to the hospital for treatment. The frequency and method of the testing depend on the spread of the virus and the risk level of the community. In the case of the Delta virus outbreak at Nanjing Lukou Airport, due to the high transmission rate of Delta virus, 7 nucleic acid tests were carried out in some areas of Nanjing. The sampling method is based on the level of risk: single sampling in high-risk areas, 5-person mixed sampling in medium-risk areas, and 10-person mixed sampling in low-risk areas (43). The community will independently complete the following tasks to cooperate with the CDCP for testing: (1) Requisition of an open area as a nucleic acid test point; (2) Notify residents of the community to perform nucleic acid testing in batches; (3) Maintain the sequence of nucleic acid testing sites; (4) Confirm that everyone in the community participates in nucleic acid testing.

#### Community managed implementation: Strictly confined and material supply

The confined communities are strictly monitored by video surveillance, and there are guards stationed at key entrances and exits. To lift the confinement, it is necessary to ensure that no new cases of COVID-19 have been added for 14 days, as described in Table 1. In order to meet the material needs of community residents, the community has designated some professional social workers and volunteers to purchase materials and distribute donated materials (31, 36). Residents who are to use mobile phones to shop online can use the APP designated by the community to purchase; and for those who do not shop online, such as the elderly and the disabled, social workers will provide on-site service.

#### Monitoring and evaluation: Health code and temperature measurement management

The lifting of lockdown in the community does not mean that the pandemic is over. In order to prevent the next outbreak of COVID-19, communities generally use the health code derived from Zhejiang Province to assess individual risk levels. The data from the health code system comes from big data. It can accurately confirm the itinerary of an individual within 14 days to assess its risk level. The health code has three colors: green, yellow and red. Among them, green represents healthy people; yellow and red represent potential patients, and red includes patients (44). Those who hold the green code can move freely, and those who hold the red and yellow code must undergo quarantine for 14 and 7 days respectively. In addition to the health code management model, the community also recruits volunteers to monitor the temperature of the residents before entering and exiting densely populated places.

## Discussion

Judging from the number of infections and deaths, China's COVID-19 prevention and control deserves recognition. In the later stage of the COVID-19 epidemic, China's CBCM basically replicated Singapore's crisis management model for SARS, namely, using big data to track patients and potential patients (45). The difference is that China's CBCM has achieved universal coverage of prevention, detection and control with the co-operation of the community, ensuring that every patient can be accurately discovered, so as to reduce the number of infections to zero. However, the secondary hazards and applicability of China's CBCM are worth considering.

### Secondary hazards of china's CBCM

China's CBCM will lead to secondary hazards such as privacy violations, heavy workloads for medical staff, financial pressure on local governments and unemployment of migrant workers.

#### Privacy violations

Due to the large differences in management level among provinces and cities in China, some communities and local CDCPs with lower management levels have leaked personal epidemiological investigation information due to improper management, causing these people to be intimidated and threatened, such as: personal information of closed contacts and sub-closed contacts in Shenyang was leaked, leading to mental health issues (46); the personal information of people returning from Wuhan to their hometown was leaked and their personal information was circulated on the internet more than 28 million times, causing these people to be harassed and discriminated against (47). In addition, in order to increase public credibility, some local CDCP have attached the personal information of patients and potential patients when publishing itinerary information, such as: surname, gender, age, and address, which makes it easy for other people in the same community to recognize them and causes discrimination against their family members and relatives (48).

#### Heavy workloads for medical staff

After identifying the communities where the patients, close contacts, and sub-close contacts are located, the CDCP will conduct nucleic acid testing on all residents in the town-level administrative areas where these communities are located. Nucleic acid sampling is usually done in 1–2 days. With the emergence of the Delta variant, the administrative area of nucleic acid testing was transformed from town-level to city-level, and the number of testing in some cities was more than six times (49). According to the “Notice on Further Strengthening the Organization and Management of National Nucleic Acid Testing” issued by the National HC in August 2021, for city-wide nucleic acid testing, when the city's population is <5 million, the testing should be completed within at least 2 days; If the city's population exceeds 5 million, it should be completed within 3 days (50). Obviously, the workload of medical staff is quite heavy. High-intensity work tasks caused some medical staff to faint during the sampling process (51). In addition, a large number of nucleic acid samples to be tested also bring tremendous work pressure to the doctors in the laboratory.

#### Financial pressure on local governments

Nucleic acid testing for all residents in town-level or city-level administrative areas will consume a lot of medical resources, especially if repeated tests were required. The Delta variant in Liwan District of Guangzhou in May 2021 alone led to more than 16 million tests in Guangzhou (52). In addition, the COVID-19 epidemic that broke out in Nanjing Lukou airport in July caused the cumulative number of tests in Nanjing to reach 40 million (53). According to the China Medical Security Administration (CMSA)'s quotation for nucleic acid testing, the price for a single sampling test is 60 yuan per person, the price for a 5-person mixed sampling test is 30 yuan per person, and the price for a 10-person mixed test is 15 yuan per person (http://www.nhsa.gov.cn/). Take Nanjing's nucleic acid testing in July as an example, which cost at least a total of 600 million yuan, which accounted for 42.46% of the funds allocated to fight the COVID-19 epidemic by Nanjing in 2020 (http://ybj.nanjing.gov.cn/). A large amount of testing fees will put heavy pressure on the financial budget of local governments.

#### Unemployment of rural migrant workers

Lockdown has a great impact on work, especially rural migrant workers (54). For employees of China's state-owned enterprises and institutions, wages and subsidies will be paid normally during the epidemic; while for rural migrant workers, they have completely lost their source of income. These workers account for about two-thirds of the employment in the secondary industry, and about half of the employment in the tertiary industry. They are highly concentrated in labor-intensive industries such as manufacturing, food services, retail services, and self-employed small businesses, all of which have been severely damaged by the epidemic (55). Some studies have shown that the two-month lockdown in the early stages of COVID-19 in China resulted in 50 to 80 million unemployed rural migrant workers (56, 57). Since then, COVID-19 caused by overseas exports has also caused some cities and towns to be locked down for about a month, which has kept the work of rural migrant workers in an unstable state. For example, Ruili (one of the cities in Yunnan, China) has locked down four times a year, with a cumulative time of more than 7 months (58).

### Applicability of china's CBCM model

China's CBCM model provides an idea for the rest of the world to follow, but whether it is universally applicable is worth considering because China's CBCM model relies to a large extent on the extreme power of the government and specific cultural background.

#### The extreme power of the government

The power of the Chinese government extends to politics, culture, education, religion, economy, social life and other fields, which penetrates into the whole society and affects every citizen (59). Its legitimacy is to convince people that the government is doing good things for the collective benefit (60). Therefore, the Chinese government can deploy any social resources in the name of protecting people's lives (61), which includes: unconditionally obtaining customer information from enterprises, shopping platforms and social platforms, and applying it to tracking and positioning (39); dispatching a large number of medical resources and medical staff across provinces and cities to conduct nucleic acid testing for all residents in the town-level or city-level (36); unilateral controlling of public opinion to ensure the smooth progress of CBCM (62); conducting legal sanctions on residents who do not cooperate with China's CBCM model (42).

#### Specific cultural background

The obedience to Confucianism since ancient times, coupled with the introduction of modern Marxism, has resulted in the specific cultural value unique to China, namely submissive society and collectivism. Confucius' idealistic social assumes that people with higher moral and ability levels have higher social ranks, which give higher-ranking people more moral superiority and legality of rights, so their subordinates should obey and cannot challenge their authority (63). In the long run, China's submissive society has been formed. In this social model, people are accustomed to gaining a sense of security by obeying the strong government control, such as receiving multiple nucleic acid tests and restricting travel. This is why the greater the control of the Chinese government, the higher the satisfaction and support of the Chinese people with the government (64). In addition, the repeated success of the zero-tolerance approach has to a certain extent confirmed the superiority of a submissive society and strengthened people's belief in government. The localization of Marxism in China makes collectivism regarded as the ultimate morality of society and energy for generating productivity (65). Palko believes that collectivism is essential for China to successfully control the pandemic (41). Collectivism emphasizes the interests of the group over the ego. Groups who identify with collectivist values tend to find common values and goals (66). In order to achieve the common goal of anti-epidemic, Chinese people generally believe that being kept at home and co-operating with the government's unified management system is a manifestation of safeguarding group interests (36). Therefore, Chinese people are highly co-operative.

## Conclusion

This article defines China's CBCM model, namely, a community-wide process in which the government, ministries, community organizations and community residents jointly manage the driving factors of crisis, eliminate it and monitor it. Among them, the government is responsible for formulating a unified prevention strategy, mobilizing resources and information disclosure, while the community is in charge of implementing government policies according to its own situation. China's CBCM basically replicates Singapore's crisis management model for SARS and with the co-operation of the community, has achieved universal coverage of prevention, detection and control of COVID-19. However, China's CBCM model does have secondary hazards such as privacy violations, heavy workloads for medical staff, financial pressure on local governments and unemployment of migrant workers. At the same time, it largely depends on the extreme power of the government, a submissive society and collectivism. It is worth noting that some potential drawbacks, such as protests or attempts at civil resistance against the management may also occur in the process of CBCM. Attention to these issues is necessary because it affects the efficiency of CBCM.

However, following Singapore's successful response to SARS, China's CBCM model once again confirmed the importance of the government's leadership in responding to major crises (67, 68). When a crisis breaks out, it is not enough to rely solely on the free response of the market and the community itself, because a profit-driven market has almost no motivation for co-operation and complete community autonomy will lead to management chaos. As a result, communities compete for material and medical resources and private hospitals refuse to admit the poor. Such phenomena occurred in the early stages of COVID-19 in China before the central government intervened (1). China's CBCM model, namely the government-centered and community-cooperative management model, can be used to overcome the shortcomings above.

Moreover, “flattening the curve” is a common prevention and control strategy used during the COVID-19 pandemic (69). This strategy reduces the infection rate by increasing social distancing and reducing social spheres to ensure that medical resources can afford existing patients. In this way, more time can be obtained to develop vaccines and achieve herd immunity (70). However, how to effectively ensure that medical resources are sufficient is a difficult problem, especially when the virus continues to mutate. At the same time, the continuous mutation of the COVID-19 virus poses new challenges to the development of vaccines. After weighing many uncertain factors, China has adopted the zero-tolerance approach. Such an approach can certainly ensure that there are adequate medical resources, but is essentially an excessive anti-pandemic strategy.

## Data availability statement

The original contributions presented in the study are included in the article/supplementary material, further inquiries can be directed to the corresponding author.

## Author contributions

Conceptualization and formal analysis: ZS and MW. Data curation and investigation: ZS. All authors contributed to the article and approved the submitted version.

## Conflict of interest

The authors declare that the research was conducted in the absence of any commercial or financial relationships that could be construed as a potential conflict of interest.

## Publisher's note

All claims expressed in this article are solely those of the authors and do not necessarily represent those of their affiliated organizations, or those of the publisher, the editors and the reviewers. Any product that may be evaluated in this article, or claim that may be made by its manufacturer, is not guaranteed or endorsed by the publisher.
